# Towards gender-transformative metrics in seed system performance measurement: insights for policy and practice in Sub-Sahara Africa

**DOI:** 10.1186/s43170-024-00291-6

**Published:** 2024-09-27

**Authors:** Eileen B. Nchanji, Odhiambo C. Ageyo, Ranjitha Puskur, Noel Templer, Enock K. Maereka

**Affiliations:** 1 International Center for Tropical Agriculture, Nairobi, Kenya; 2 International Rice Research Institute, New Delhi, India; 3 Seed System Consultant, Harare, Zimbabwe

**Keywords:** Gender, Seed system indicators, Performance indicators, Gender frameworks, Empowerment, Food security, Income, Policy

## Abstract

**Context:**

Food insecurity in Sub-Sahara Africa hinges on addressing salient gender inequities within the seed system. While efficient seed system promises reduced systemic inefficiencies to fast-track seed delivery to the smallholder farmers, a dearth of standardized industry metrices to understand the intersectionality of seed system and gender issues exist. Specifically, metrices on guaranteed seed access, reach, benefit, women’s empowerment and ultimate transformation of women, youth and vulnerable people’s livelihoods are less understood. The existing metrices are aggregated at very high levels and limit the ability of policymakers and industry stakeholders to effectively address gender-based inequities for an optimized seed system.

**Objective:**

Our objective is to challenge the *status quo* industry metrics used by seed industry players and apply a gender framework that strikes a balance between the needs of women, youth and vulnerable peoples in the system, *vis-a-vis* the need of public, private, and civil society actors. Therefore, the study seeks to evaluate how seed system metrics can be effectively tailored to address gender gaps for enhanced agricultural productivity and food security in Sub-Sahara African context. It also refines the proposals of Kennedy and Speilman and introduce gender-specific metrices that may hold promise to address women and youth’s challenges within the seed system.

**Methods:**

A systemic review of current industry metrices was conducted and the newly developed reach, benefit, empower and transform (RBET) framework was applied to synthesize the responsiveness of current seed industry indicators on gender issues. Online databases and repositories with key search words that returned 204 results including some gray literature.

**Results and conclusion:**

Using common bean seed system as an illustration, the study found critical gaps in measuring seed industry performance, innovation, structure, seed registration and quality control, intellectual property rights using the reach, benefit, empower and transform approach. Thus, a set of gender responsive indicators was suggested to address gender and inclusive matrices that the seed industry often neglects. Using the reach, benefit, empower and transform approach we have included gender responsive indicators meant to close existing gender gaps. Some of these indicators addressed include women participation, trait preferences, seed packaging sizing, seed system leadership, decision-making capacities, labor intensity/drudgery and use of digital platforms such as point-of-sale tracking systems to reach last mile farmers among others.

**Significance:**

This study uses the newly–developed Reach, Benefit, Empower, and Transform (RBET) Framework together with the already existing Spielman–Kennedy framework. It is timely to inform policymaking process on seed system design, to enhance seed industry performance monitoring, and provide practitioners with the knowledge and missing links in efforts to align the seed system's performance with gender outcomes in a measurable manner.

## Introduction

Agriculture is the primary economic activity in many developing countries, employing nearly half of the Sub-Saharan Africa’s working-age populations, of which women, youths and other vulnerable groups are disproportionately represented (FAO 2021). The sector is also riddled with systemic challenges and structural inefficiencies despite numerous steps taken towards improving crop productivity and reducing hunger and malnutrition for the rapidly growing population. One critical pathway for improving productivity is establishment of a robust and gender responsive seed system that delivers improved seed varieties tailored to the diverse needs of client groups, particularly women, youths and the vulnerable individuals. These seeds also need to have high-yielding potential, be market relevant and resilient to climate change regimes such as drought. The significance of this approach stems from the intersectionality of gender roles with the needs of these groups. For example, some innovations that include delivery to last miles and using point-of-sale tracking apps have achieved seed access in remote areas, but the persistent challenge remains on specific metrics that may be used in the seed system to address peculiar traits of women and youth differentiated requirements (Ojiewo et al. 2018a, b). Therefore, the study seeks to evaluate how seed system metrics can be effectively tailored to address gender gaps for enhanced agricultural productivity and food security in Sub-Sahara African context.

From the above discussion, it appears that there are significant industry-level improvements, but reliable metrics that can be used to measure seed is understudied (Spielman and Kennedy 2016a). Perhaps, there is an urgent need to relook at robust seed system metrics that apply gender-responsive frameworks and within crops that appeal to women and youths such as common beans (see Nakazi et al. 2017; Nchanji et al. 2021a, b; Ojiewo et al. 2018a, b). As a positive outcome, some new framework that tracks whether these vulnerable demographics are reached, whether they benefit, empowered and have achieved sustainable transformation, i.e. Reach, Benefit, Empower, and Transform (RBET) framework has been developed (Puskur et al. 2021). However, limited research exists on how to align some of the previously developed seed system metrics with the RBET framework and particularly with regards to gender issues. Besides, new industry metrics on seed system functions, actors and seed security need to be integrated for a robust seed system (McGuire 2007; Sperling et al. 2020a, b).

Traditionally, seed systems fall into two main categories, namely; the formal and the informal seed system depending on the involvement or absence of official regulatory authorities (Abdi and Nishikawa 2017; McGuire and Sperling 2016; Sperling et al. 2021). The formal seed system is ladened with systemic inefficiencies and weak oversight (system "corruption") (Kapran et al. 2021; Wineman et al. 2020). The informal seed sector predominantly has low-yielding, genetically inferior germplasm that results in suboptimal agricultural productivity. In recent years, however, an intermediate seed system has emerged to bridge the gap between the two and promises access to quality seed for farmers at last miles within the informal set-up and at lower costs (Argaye 2019; Louwaars and De Boef 2012). In the context of crops with limited commercial viability such as common beans (the primary focus of this study), the intermediate seed system plays a critical role in bringing seeds to the communities. Despite intermediary seed system’s promises, the informal seed system is still the most dominant seed access method in most developing countries, supplying more than 90% of the farmers’ seed needs in Sub-Saharan Africa (Mekbib and Deressa 2016; Paudel et al. 2013; Shiferaw et al. 2010; Sperling et al. 2020a). On the other hand, formal channels supply less than 3% of the seeds in Sub-Saharan Africa (Sperling et al. 2020a).

Over the years, various studies have highlighted the benefits of each system and for various crops (Bishaw et al. 2012). Other studies have highlighted the need for coexistence and synergy between the three seed systems to ensure delivery at scale for women-preferred legumes such as common beans (Maereka and Rubyogo 2020). However, these mixed models involve enormous complexity in assessing performance to help stakeholders improve access to seeds. The recently developed seed access indices and seed system performance indicators and associated system category classifications serve as a blueprint towards better metrics but they require innovative ways of integrating other new seed system indicators such as seed actors and seed security (Spielman and Kennedy 2016b). The main challenge is gathering the required information at the appropriate time to support decisions and policy and having a system that collects and analyses this information in a cost-efficient manner and on a continuous basis. Several indices, such as the African Seeds Access Index (TASAI), access-to-seed index, and SeedSAT, among others, have also been proposed, but these indices rely only on formal seed system metrics, which are often highly aggregated with no gender indicators (Mabaya et al. 2021).

These "easy to obtain" and highly aggregated indicators, such as seed demand and supply, guide industry players and policymakers on the seed industry's performance; however, they offer little analytical value because only broad assumptions and conclusions may be drawn from them (Spielman and Kennedy 2016b). Using these general aggregated indicators is a sharp contrast to modern day consumer business models that seek a better understanding of diverse customers, their preferences, and their experiences at value chain touch points. Local-level indicators such as the number of farmers (and their demographic characteristics such as age, gender and race or socioeconomic characteristics such as landholding and income) accessing seeds of a specific variety in a given season are rarely and/or poorly captured. Current seed system performance indicators are incapable of capturing the dynamics of gender (women, men and youths—young men and young women) in seed access. There is a need to develop and explore how these indicators measure how and who seeds reach, benefit, empower and transform the livelihoods of farmers, especially women and youths.

While Spielman and Kennedy (2016a) developed several seed system performance indicators, gender was never considered, and this is a gap that urgently needs to be filled. This study is also the first to assess such indicators in line with the Reach, Benefit, Empower, and Transform (RBET) Framework developed by Johnson et al. (2018), Kleiber et al. (2019) and Nchanji (2022) integrated in the already existing Spielman–Kennedy framework. This approach will provide practitioners with the knowledge and missing links in efforts to align the seed system's performance with gender outcomes in a measurable manner. Thus, the study seeks to evaluate how seed system metrics can be effectively tailored to address gender gaps for enhanced agricultural productivity and food security in the Sub-Saharan African context.

### Gender roles in common bean seed systems

As alluded above, common bean (*Phaseolus vulgaris* L.) is regarded as a "women's crop" due to women’s preeminent contribution to its production and processing activities at the smallholder subsistence level (Mugisha et al. 2019; Nakazi et al. 2017). For example, women have been reported to contribute up to 90% of the labor in common bean plots in Malawi and have greater access to bean varieties; thus, they have improved decision making concerning such varieties (Njuki et al. 2011). Other findings, such as those of Mujaju et al. (2017), concur that at the household level, women are more likely to make decisions on multiple crop varieties to grow, field sizes, and field allocations. Similarly, in Cameroon, women carry out bean production operations, while men engage in regional and international marketing of the produce (Siri et al. 2020). A recent study on the yellow bean corridor in Tanzania showed women's involvement in the upper/lower echelons of the bean grain retail trade, but their participation was more likely to be limited by their localities and type of trade (export or local aggregation) (Kalb 2020). Additionally, women dominate the small informal seed markets in most rural towns (ISSD 2013). Puskur (2021) indicated that most projects reach women, but few benefit or get empowered from them. Even though access to seeds and training in seed production sometimes improve women’s social status, this does not necessarily lead to their empowerment.

Despite these contributions, societal norms and values have also historically limited the participation of women in the governance of seed systems (Adam and Muindi 2019; BenYishay et al. 2020; Ojiewo et al. 2018b; Puskur 2021; Siri et al. 2020). It can be anticipated that the formal seed system should overcome such longstanding and ubiquitous inadequacies by involving more women in the formal seed system through transformations led by women (African Biodiversity Network and GAIA 2015). The critical contribution of women and youths in creating pathways to achieving food and income security has recently been recognized as a strong indicator of seed system performance (De Schutter 2010; Galiè et al. 2017). Women, youths and other disadvantaged groups are being empowered through active participation in seed system governance via participatory plant breeding (PPB) and participatory varietal selection (PVS) activities that address varietal and trait preferences, especially for women (Karimi et al. 2019; Mukankusi et al. 2018; Ojiewo et al. 2018b). These approaches serve as a paradigm shift in ensuring that women’s varietal preferences are considered in breeding that translates to a gender-responsive seed system. The newly developed triadic comparison of technology options (tricot) approach further promises lowered costs compared to the two approaches, easy to scale-up and promises to identify and integrate consumers’ preferences and market demands (de Sousa et al. 2024).

Varietal attributes such as seed color, seed size, maturity time and cooking time are said to be more appealing to women than to men (Abate et al. 2018; Puskur et al. 2021), even though men and women often want the same traits to be prioritized differently (Nchanji et al. 2022a, b; Tufan et al. 2018; Weltzien et al. 2019). PPB offers highly localized instant access to seeds of preferred varieties, while other approaches, such as demand-led breeding (DLB), are expected to disseminate seeds at scales beyond the testing localities. Like in the case of PPB and PVS, DLB is recommended for combining local traits, varietal preferences and market insights from various value chain actors (Mukankusi et al. 2019). However, DLB requires re-thinking how to integrate gender as a political strategy through which crop breeders and donors, not only hold much decision-making power but let in gender specialists to be at the forefront in informing the women and youth needs. (Tarjem et al. 2023).

However, DLB may not provide precise avenues for assessing spatial seed system performance and outcome comparability across genders. Recent women’s empowerment approaches, including gender-responsive PVS approaches, gender-responsive demand-led breeding approaches and gender + customer and product profiles, have been designed to sporadically generate evidence on gendered breeding (Nchanji et al. 2021a, b, 2022a, b) that contributes to gendered seed system performance. These new approaches require modified or new indicators that measure how seeds reach women and youths, how they benefit and empower them, and how they transformational capacity (breaking gender norms and cultural and institutional barriers)(Beck 2007; Malapit et al. 2020). This is particularly important for crops that women prefer, especially legumes such as common bean, which are not well studied or reported in mainstream literature.

### Common bean seed systems in SSA

This research focuses on common beans, a women-preferred legume in Sub-Sahara Africa (shortly-discussed) and also a the focus crop of the Pan-Africa Bean Research Alliance (PABRA) (Buruchara et al. 2018; PABRA 2018). The study acknowledges the limitations of focusing on a singular crop while it could potentially benefit the heterogeneity in seed system and nuances on open-pollinated varieties. Despite this “loss”, the concentration on common beans is strategic and the findings are generalizable given that the seed industry players sometimes deal with more than one specific crop (Cromwell et al. 1992). Notably, the Pan-Africa Bean Research Alliance (PABRA) works across 31 countries with National Agricultural Research Systems (NARS) partners in SSA and has systematically developed and released improved common bean varieties using conventional and market-responsive seed systems (Buruchara et al. 2011). It promotes gender-responsive approaches, seed system metrics and seed delivery approaches such as seed credit models, seed revolving funds, digital payment solutions, and use of small packs (2–5 Kgs) for women and youths with limited capita (Onyango 2020). Similarly, the RBET framework is developed within the CGIAR system, which deals with other legumes, among which common beans are valued. Briefly, the RBET framework assesses how agricultural development investments and interventions empower women to stimulate rural revitalization and promote women's empowerment (Quisumbing et al. 2019). In the seed system, the framework reflects how the seed industry is positioning its products within the seed value chain to respond to men, women and youth needs across the value chain. Also, CGIAR initiatives such as SeedEqual seek to address demand and supply challenges in the seed sector, with proposals of reaching more than 2.5 million women producers with high-yielding, climate-smart and fast cooking crops and cereals and legumes such as common beans (CGIAR 2021). The idea is to revolutionize the thinking and implementation of gender issues and the transformation of livelihoods within the oneCGIAR initiatives.

Furthermore, common bean seed systems are underdeveloped in SSA (Maereka and Rubyogo 2020). Seed companies often perceive legume seeds, in general, as low business products due to inconsistent repeat sales because farmers use informal channels to access legume seeds and because they are open-pollinated crops (Birthal et al. 2011). Despite recent government incentives to encourage private sector investment in the legume seed business in general, only a limited number of private companies cultivate common bean seeds in SSA. For instance, PABRA seed expert data indicate that countries with no more than three private seed companies are Zimbabwe, Mauritius, Madagascar, the DRC and Zambia, as shown in Table 1, out of 11 countries (Rwanda, Uganda, Madagascar, Kenya, Mauritius, Tanzania, the DRC, Lesotho, Malawi, Zambia and Zimbabwe). For example, only 3% of the bean growing areas in Kenya use certified common bean seeds (Kariuki 2015).

**Table 1:** Countries with small and medium seed companies that also produce bean seeds within PABRA-targeted countries

Country	Small and medium seed companies
Rwanda	12
Uganda	15
Madagascar	3
Kenya	7
Mauritius	3
Tanzania	9
DRC	2
Lesotho	4
Malawi	8
Zambia	2
Zimbabwe	3
Total	68

Seed companies have therefore invested very little in understanding and developing the bean seed value chain, especially when compared to the maize subsector. There is often a mismatch between a private company’s seed production and supply and between its seed production and farmers’ varietal preferences. This disconnect often stems from the focus on farmer and breeder’s varietal and trait preferences (Abate et al. 2011), not including preferences of other value chain actors such as traders, processors, consumers, etc. (Nchanji et al. 2022a, b). Such highly aggregated findings often obscure gender aspects, which are key ingredients in evaluating seed system performance. Even though this has improved in recent years, especially with the gender-responsive demand-led breeding approach, it is still very low for a continent where legumes form the central part of staple diets (Bosch et al. 2017).

It is within these confines that this study evaluates seed system responsiveness to gender, and it applies the RBET framework to further build on Sperling-Kennedy seed system performance metrics.

### The evolution of gender-responsive seed system performance indicators

Historically, seed volumes produced and sold have been used as indicators of seed sector performance (Cromwell et al. 1992). This is associated with bias in reporting due to highly aggregated and minimal reporting of socioeconomic welfare indicators for seed users. However, there is a consensus that seed system performance indicators developed on an ad hoc basis serve only specific evaluation purposes (Spielman and Kennedy 2016b). The identification of holistic indicators that cater to seed systems and include gender dynamics has been slow in development and has evolved over time since the proposal of Costanza et al. (1992) in the field of environmental management. The adoption of their work in a Ugandan workshop, as expressed in Sperling (2001), perhaps paved the way for conceptualizing the components of a seed system within farmers‘ seed systems, including propositions of some generic indicators. At that time, it was perceived that direct measurement of smaller parts of the seed system through indicators such as pricing was more precise, easier, relevant and fast to model or integrate. It was also important to move beyond the farmer and investigate seed system properties with components such as equity in seed access with moderate precision. In their final analysis, Sperling (2001) argued that evaluating the value of a seed system in terms of evaluating its overall performance or “health” is important but less precise, difficult to measure, less relevant and cumbersome to model or integrate. The other seed security literature has focused on four priority areas, namely, seed availability, quality, access, use and control, to elaborate on the gender dynamics of the seed system (FAO 2016). These parameters are based on the Food and Agricultural Organization’s definition of seed security and only indicate how the supply and demand dynamics of seed systems address these four issues (*ibid*). Thus, the subsequent work of Spielman and Kennedy (2016a) seems very relevant because it traces a pathway of well-researched and easy-to-integrate methods that would be relevant for measuring seed system performance.

Contemporary developments have witnessed rejuvenated efforts and attempts to redefine the seed system metrics, with a focus on gender issues. One such framework focuses on seed actors such as farmers, producers, government agencies and other private sector players, seed system function (from variety development to how it drives the food system outcomes) and food security that focuses on seed access, availability, quality and varietal suitability among others (RTB 2016; Westengen et al. 2023). While the framework is at its development stage and may be valuable to add to other frameworks, this study focuses on the CGAIR RBET framework but integrates important elements of the above framework. The recent development of the RBET framework as a tool for evaluating gender responsiveness in CGIAR programs and projects provides a pathway for building evidence on gender dynamics within seed systems (Puskur et al. 2019). While Spielman and Kennedy (2016a, b) provided an avenue for quantifying, tracking and reviewing seed systems and seed industry responsiveness to end-user needs, the conventional but ad hoc household survey data and the data generated by seed companies, distributors, retailers and experts such as breeders have been systemized with the shortcoming that data are very limited or difficult to collect in most cases due to missing information on indicators of focus.

Several sources of data that track seed system performance indicators have been provided by the Consultative Group on International Agricultural Research (CGIAR and NAR partners for 20 crops in more than 30 countries (CGIAR DIIVA 2021) (Table 2). Seed system performance indicators are often intertwined with breeding indicators because breeding leads to better quality seeds that are sold to smallholder farmers. Previously, several of the indicators used in measuring seed system performance were developed from an integrated breeding and seed system approach; these indicators were year of variety release, years for the first to last variety released in the country, the number of breeders and the number of varieties released thus far. However, these datasets do not contain gender information. For example, they do not reflect the number of varieties that women could access, use or benefit from after adoption.

**Table 2:** Common bean seed system indicators and data on varieties. *Source*: Author's computation based on the CGIAR DIIVA (2021)dataset

Country	First-last release year	Number released	Average geographical area (Ha)	Number of full-time equivalent (FTE) breeders	Year data collected	Source
Burundi	1979–2010	35	2	0	2009	Expert opinion
Congo	1985–2009	35	2.3	2.8	2009	Expert opinion
Ethiopia	1973–2008	32	4.1	4.8	2009	Expert opinion
Malawi	1995–2009	15	6.1	4	2009	Expert opinion
Mozambique	1986–2010	15	3.3	2.1	2009	Expert opinion
Rwanda	1970–2010	61	0.61	5.1	2010	Survey
Tanzania	1980–2010	29	4.2	2.5	2009	Expert Opinion
Uganda	1968–2010	18	2.2	3.3	2010	Survey
Zambia	1985–2014	30	2	1	2015	SCCI Variety Register

CGIAR DIIVA elicits expert opinion (people with experience in the subject matter) with the verification of the accuracy of these estimates with specific and rigorous adoption studies

Additionally, these datasets are aggregated at the national level. The data are missing from the private industry and other seed value chain actors, such as seed companies, distributors, community seed banks, donor-funded seed providers, and emergency seed providers. Nonetheless, such databases are important for evaluating and tracking some indicators that can be used to measure seed system performance.

Despite these efforts, little is known about the real-time indicators that measure how women, men and youths are reached and how they benefit, are empowered, and transformed by the seed system and vice versa. While sporadic data exist on the number of women affected at several small, localized levels, nationwide data are lacking on how the seed industry tailor-makes its products to cater to women's needs. There is an urgent need to develop quantifiable indicators that show the performance of the seed system and how it responds to gender issues such as women's empowerment within the RBET framework. Indeed, the lack of data in these areas in seed systems was highlighted in Puskur et al. (2021).

In this study, we review several of these indicators proposed by Spielman and Kennedy (2016a, b) modify them and add new ones to fit within the RBET framework. Using this framework, we developed gendered performance indicators that provide a realistic and measurable way of reviewing how the seed system addresses gender issues. The RBET framework is augmented by performing a systematic review to find studies that report some gender elements in seed systems and how they fit within both the RBET and Spielman and Kennedy (2016a, b) proposed framework. Such a review is important for generating evidence on industry practices to quantify how the seed system responds to gender needs or a lack of responsiveness. This study contributes to the literature by reviewing studies that have reported indicators that quantify seed system performance with respect to gender and developing gender-responsive seed system performance indicators.

## Methods

### The reach, benefit, empower and transform (RBET) framework

**Fig. 1:**
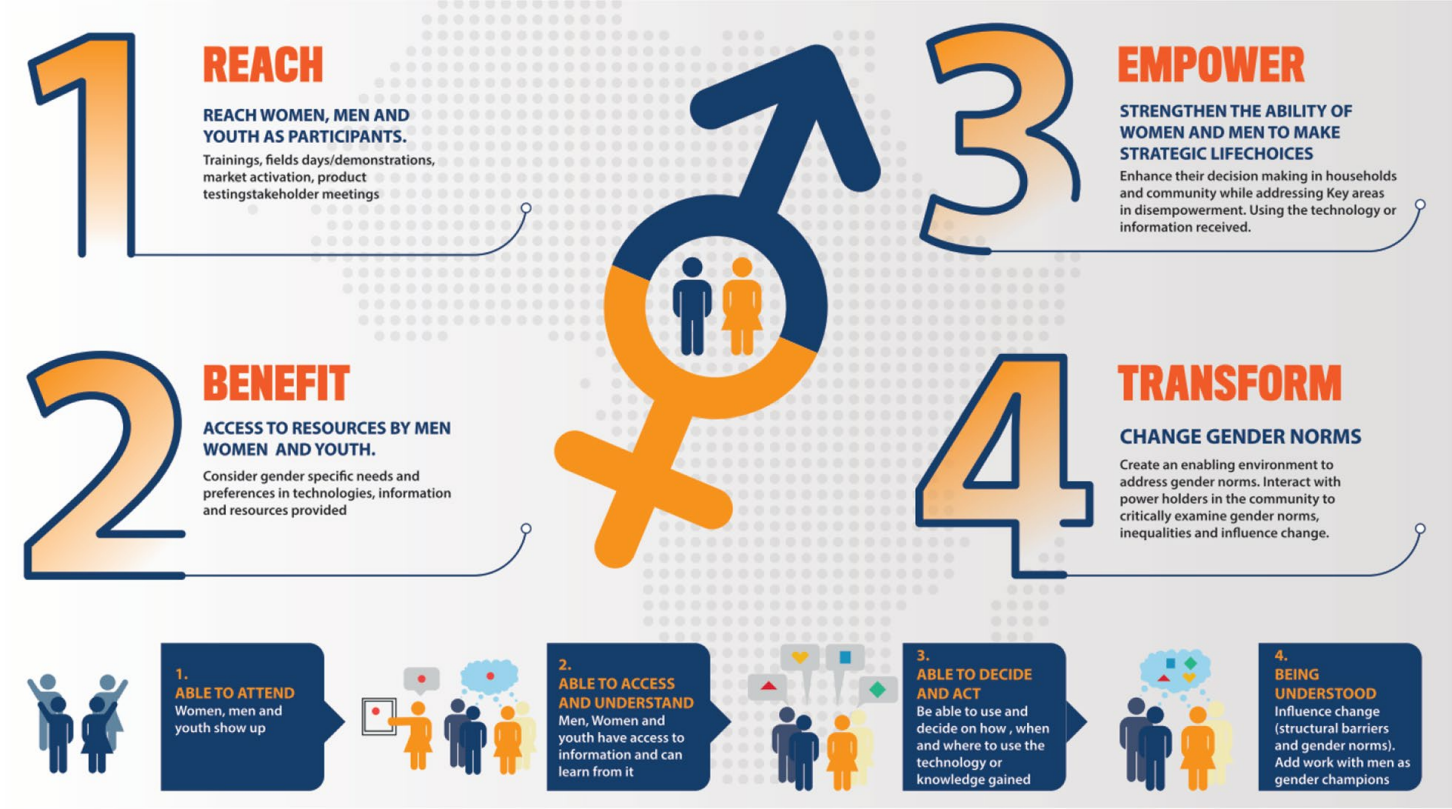
Reach, benefit, and empower frameworks (Nchanji 2022)

According to Johnson et al. (2018), Kleiber et al. (2019) and Nchanji (2022), the RBET framework aims to classify projects in terms of their gender approaches and evaluates whether they are designed to reach, benefit, empower and transform the situations of women, youths and men. It reflects the evolution of gender integration and practices over time and is very consistent with empirical evidence on transformative gender approaches in development-oriented projects. The reach domain aims to identify women, youth and men’s participation through training, demonstrations, markets, and product testing, among other activities, as shown in Fig. 1. The benefits domain aims to evaluate access to resources and consider gender-specific needs and preferences in technology, such as improved variety adoption and resource use efficiency. The empower domain looks at gender-strengthening interventions that enhance decision making within households and at the community level, especially for women and youths, while addressing gender-disempowering activities and issues. Finally, the domain of transformation aims to move beyond just empowerment and into changing gender norms by creating an enabling environment that addresses norms and taboos and culturally underpins gender-disempowering activities and issues (Nchanji 2022).

The approach of the present paper is to use the RBET framework and evaluate how it fits within the seed system performance indicator matrix developed by Spielman and Kennedy (2016a, b). This provides a wider angle for re-examining the propositions of Spielman and Kennedy (2016a, b) to develop a new matrix that integrates seed industry domains such as industry performance, innovation, structure, intellectual property rights and regulations into gendered and measurable units. For example, under the reach domain, one may want to measure how industry performance indicators such as the number of seeds sold, seed packaging, and seed prices correspond to gender issues. The critical question may be whether men’, youth’s and women's seed access and preferences differ across different packaged quantities and whether there are any price differences between the genders. Critically examining and attempting to answer such questions will help develop new seed system measurement metrics that address both industry performance and gender. Another example is when tracking how seed industry structure, measured by the Herfindahl–Hirschman Index (HHI) and CR4/8 (four and eight-firm concentration rate), corresponds to seed market/distribution concentration and if the seed industry structure caters to gender differences in terms of reaching farmers (Deconinck 2019). This approach may provide a way of evaluating how seed distribution systems, seed companies and other industry players configure their reach to men, youth and women in rural‒urban settings. Finally, it may be interesting to provide evidence on how the number of intellectual property rights or seed regulations affects women's access to and rights to purchase, use and control seeds. Developing a seed system metric would help seed regulatory authorities establish policies that address women’s seed access rights. The list is not exhaustive but gives examples of a dire need to reassess the Spielman–Kennedy seed system metrices and propose gender-sensitive metrices that are applicable within the seed system.

### Systematic literature review and search criteria

The Preferred Reporting Items for Systematic Reviews and Meta-Analyses (PRISMA) is a standard tool used for systematic reviews and meta-analyses (Page et al. 2021). This approach was applied here to select studies from two major repositories of interest. In this case, only a systematic review and not a meta-analysis was conducted. The data were collected from publications from 1990 to 2022. The first was a quick Google Scholar™ search with the following phrases: "*gender" AND "seed system" AND “seed system indicators”* AND *"common beans" OR "Phaseolus"*. This process yielded 167 results. The second search used an all-in-text search with the phrases: *allintext:"gender" AND "seed system" AND "common beans" OR "Phaseolus* AND “women”. It yielded 33 results among which the previous 167 results were part of. Another search was conducted on the CGIAR repository with the search phrases *"seed system" AND "gender" AND "indicators" AND "common beans" AND [publication date: 01/01/1990 TO 12/31/2022],* which yielded only 28 results. We purposively included a few gray studies (documents produced by organizations with non-commercial publishing), especially reports from the PABRA and CGIAR Gender Platforms, as the basis for our research. By searching for gray literature, we maximized the comprehensiveness of our literature and mitigated publication bias, as discussed in Gusenbauer and Haddaway (2020).

### Study selection, inclusion, and exclusion criteria

**Fig. 2:**
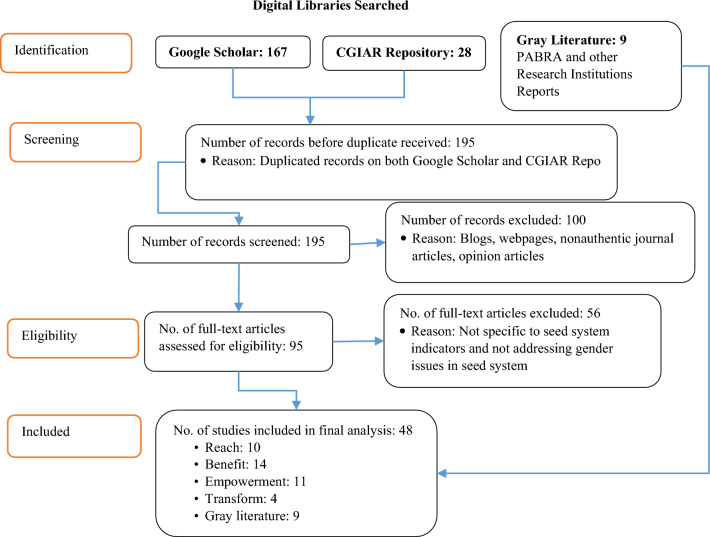
Flow chart indicating studies included in the systematic review of seed system indicators. *Source*: Author’s representation based on (Moher et al. 2009) PRISMA guidelines

Peer-reviewed journal articles from reputable journals and reports from other organizations promoting common bean in Sub-Saharan Africa were included based on the following criteria: (1) the study reported on legumes with a focus on common beans; (2) the study attempted a disaggregated analysis, preferably by gender; (3) it was written in English; and (4) it was a review or instructive article. Blogs, web pages, opinion pieces, and magazine articles were excluded due to a lack of scientific rigor. A total of 36 (including gray literature) of the 195 studies were included in our final analysis. The data extracted from each article included the author’s name, year of publication, study interventions, gender issues addressed, and seed industry indicators, among others. The summarized documents detailing the seed system and gender indices used are shown in the section summarizing the results and conclusions (Fig. 2).

## Results

In this section, the current study examines current attempts to assess seed system performance with respect to gender by using literature that defines some of the proposed indicators. By considering peer-reviewed evidence and the ongoing policy discourses around SSA seed industry development, this study provides examples of the deliberate use of the seed system in order to include gender dynamics in terms of the Reach, Benefit, Empowerment and Transform frameworks (for women, men and youths). It also aims to highlight the shortcomings of key indicators that industry players use in different countries. In the discussion section, the study discusses what evidence is missing or a set of alternative indicators that may enhance innovative approaches that make seed systems gender responsive.

### Reach

In the reach domain, strategies for reaching women through capacity development and metrics showing participation in seed systems (such as training and meetings), access to seeds, and extension services were evaluated (Johnson et al. 2018; Nchanji et al. 2022a, b). To track these participation indicators, the study mapped them to measurable indicators as proposed by Spielman and Kennedy (2016a, b). First, with regard to seed access indicators, a recent cross-country (Kenya, Uganda and Tanzania) study by Marimo et al. (2021) revealed the informal seed system to be the dominant system and was accessed by 69% of women (own seeds and seeds from neighbors). In Rwanda, women were more likely to access seeds through informal channels; thus, a more cost-effective and inclusive strategy to reach them was leveraging their social networks (Vaiknoras et al. 2019). In Kenya, NGOs seem to work more with women (78%) than men (58%) in the seed system to balance the formal/improved seed access dominated by men (ibid). This implies that seeds promoted by NGOs seem to be accessed more by women because they are intentional. In Madagascar, women participate in seed production and attend demonstration plots in the same way men do, resulting in nondiscriminatory pricing that does not favor any gender; the willingness to pay is 171% (Bosch et al. 2017). However, the study showed that, irrespective of price discrimination or the free provision of seed and farming information, the willingness to pay more for improved seeds was low.

In Kenya, PABRA and the Catholic Relief Services develop and use the Point-of-sale (PoS) app to track real-time seed distribution. The goal of this study was to determine whether the PoS application could shed light on new varietal dissemination and farmers’ preferences so that seed companies and agro-dealers could use this information to improve their sales, targeting future seasons. The results showed that more males (62%) accessed more varieties than did their female counterparts. A study also revealed that high-iron bean (HIB) varieties retail between 2.20 and 2.50 US dollars per kilogram (Onyango 2020). However, there was no gender disaggregation; thus, the evidence of the effect of gender-based seed cost/pricing was inconclusive. Gender-disaggregated price indicators could be important for understanding price discrimination among men and women. Looking at the seed packaging indicator, it was found that packet sizes between 2 and 5 kg dominated the market. This was confirmed by the Tropical Legumes II program, which sold 943,170 small seed packs of common bean and other legumes in 13 African countries (McGuire and Sperling 2016). This implies that smaller seed packages may be preferable to smallholder farmers, especially women (Gichangi et al. 2012). On the other hand, Rubyogo et al. (2016) reported that 76.4% of farmers were satisfied with Malawi's 1.5 kg package, but no gender data are available to determine whether this approach works for men and women. Gender disaggregation was not presented in the study above but was presented in (Gichangi et al. 2012).

Another indicator within the reach domain is the number of niche varieties/bean products tailored toward a certain group using nontraditional methods to improve seed access and delivery. The legume seed industry in Kenya and Uganda is developing rapidly, and new biofortified varieties high in iron and zinc (*Nyota, Angaza* and *Metameta* in Kenya; NAROBEAN 1, NAROBEAN 2 and NAROBEAN 3 in Uganda) have recently been released (Ugen et al. 2021). Seed access for these varieties has been enhanced for women through the Seed Credit Model (SCM) and Community Production and Marketing System (COPMAS) (ibid). While these models have yet to be scaled up, a study in Kenya found that women have dense seed networks that could be utilized to scale the models. In addition, these networks heavily rely on "informal sources" rather than men's networks (Otieno et al. 2021). This implies that even if we decide to focus only on formalized seed models, farmer-to-farmer seed exchange (informal) should be addressed within the seed system because it is the main source/risk for misinformation. Minimizing such risks of misinformation could occur through community-led seed banks harmonized within national policies. An example is the community seed bank of Kiziba in Uganda (Wilkus 2016). This allows for the reliable transfer of seeds and other materials from the local to the formal seed sector, thus eliminating informal linkages and resulting in lower seed quality. In Ethiopia, farmer-led seed delivery organized around Local Seed Businesses (LSBs) to produce and market quality seeds that were not attractive to private companies seemed to solve the challenge of accessing seeds for women (Ojiewo et al. 2018a, b).

As proposed by Spielman and Kennedy (2016a, b), studies assessing the use of the Hirschman-Herfindahl Index (HHI), which measures firms' market shares in the seed market disaggregated by main seed/grain source for farmers, are presented in Table 4. In Kenya, the largest 4 and 8 retailers shown by the four-firm concentration (CR4) ratio (%) and eight-firm concentration (CR8) ratio (%), respectively, were 19.29% and 33.92%. Thus, the informal seed industry may be described as 'low moderately concentrated', as indicated by the HHI of 5321 in the country. The largest 4 and 8 largest wholesalers in Kenya controlled 34.76% and 59.84%, respectively, and had an HHI of 9460 (Kibiego et al. 2003). This indicates a nearly pure competitive market structure for the seeds. On the other hand, Ethiopian market concentration seems oligopolistic, as there are few value chain actors in each of the nodes, especially exporters. The HHI of the aggregators, who are at the local level, is 5582, indicating that the common bean seed/grain market is moderately concentrated, similar to retailers in Kenya (Palencia 2017). According to these analyses, women were positioned as traders in bean grains, but the seed industry was dominated by males (Kibiego et al. 2003).

### Benefits

In the benefits domain, the main aim is to highlight how the common bean seed system enhances the welfare of a bean farmer, especially in terms of hunger reduction, increased income and improved resilience among women (Johnson et al. 2018; Nchanji et al. 2022a, b). It is possible to quantify how these indicators improve women's welfare by measuring factors such as nutritional benefits, adoption practices, tracking time use, seed certification procedures, and community seed systems. First, adopting improved varieties is important for improving income and nutritional status. Farmers who buy seeds of improved varieties have been demonstrated to have better welfare (Walsh and Sperling 2019). An integrated impact-driven seed systems trial resulted in a 20% to approximately 68% increase in common bean adoption and improved seed production and delivery in Ethiopia (Tumsa et al. 2017). Farmers were able to obtain improved yields that resulted in surplus sales to cater to household expenditures.

In Rwanda, it was demonstrated that adopting iron-biofortified beans was successful because of women's variable preferences, with longer-lasting benefits for women (Vaiknoras et al. 2019). However, some studies indicate that males adopt improved seeds more than females do, as they have access to resources and lead in most household decision-making. This was demonstrated by (Wambua et al. (2018), who also found that households in Kenya where the main decision maker was a woman had lower yields.

On the other hand, a participatory breeding program in Uganda found women groups motivated by commercial goals, resulting in the diversification of their seeds (Wilkus et al. 2018). This implies that seed systems that improve incomes are desirable and may help improve community-level nutrition. Not integrating women's roles as decision makers in seed systems may result in disproportionate resource allocation and use. A study in Kenya indicated that female household heads made fewer decisions on incomes from common bean farming using improved variety seeds (Wambua et al. 2018). As such, PPB and PVS can be avenues for motivating women who want to commercialize bean production and move out of poverty, as they include gender training modules on farm and household decision-making.

While financial welfare is important, improved nutritional status and improved cognitive abilities through bean consumption using high-iron beans have been documented (Murray-Kolb et al. 2017). Through PABRA high-iron bean breeding, access to HIB seeds may change the nutritional status of women positively. In Ethiopia and Uganda, women were more involved in labor-intensive activities such as weeding and threshing but had little decision to sell grains (Abera et al. 2020; Katungi et al. 2019). Studies have demonstrated that even attending training and farm demos are limited by their radius of movement and society's definition of a 'good wife/woman' (Njuguna et al. 2016).

To benefit women, the seed system should be designed to be responsive across the value chain, offer more training and extension services, and give women more autonomy in decision-making. An example is CGIAR's Excellence in Breeding program, which recognizes real-life seed choices as tradeoffs or competitive events with other livelihood farmer needs but recognizes chain-wide trait prioritization that may help overcome such tradeoffs and incentivize farmers to buy seeds (McEwan et al. 2021). It recognizes women as leading informal seed traders and local intermediaries in the seed value chain, as they sort grains as "potential seeds" (Sperling et al. 2020a, b).

### Empowerment

In this domain, we track seed system studies in common bean value chains that measure empowering actions such as women's leadership, agency, skills and factors that inherently disempower them, such as drudgery, time use, decision-making power or control over resources. Most formal seed systems involve pro-men, who are designed to attract them as farmers who own land and as household heads (Paris and Rola-Rubzen 2019). Women are not recognized formally as seed users (Brearley and Kramer 2020). Thus, gender inequalities constrain women's access to and participation in seed-related decision-making processes. In the common bean seed system in Ethiopia, 30% of the seed producers were widowed, divorced, or separated women who lacked opportunities to network or participate in community meetings that discussed seed issues (Geleta et al. 2017). This limited their participation in leadership or vying for seed-bank leadership positions. In Zimbabwe, the design of agricultural extension services, especially training workshops, was reported to be a disempowering factor, as women were excluded from distant agricultural training despite women playing a dominant role in seed processing, preservation and storage (Matsa and Manuku 2013).

Seed systems have seen community seed banks engaging women and furthering their decision-making in accessing quality seeds. In Zambia, women were more active in variety selection at the local level (USAID/AfricaLead 2016). They also belonged to groups that made regular weekly payments that enabled them to purchase seeds. They also held leadership positions, giving them a voice in seed system activities. In contrast, in Tanzania, ownership and upper-level management of the surveyed seed companies revealed that women accounted for only 3% of the population (AGRA 2016). Women held clerical and smaller positions that did not put them in a position to voice their concerns, inherently disempowering them in seed system activities.

Apart from leadership, seed networks managed by women were found to create space for improved decision-making among women in Kenya, as women prefer to exchange seeds with other women (Otieno et al. 2021). Along the value chain, especially in terms of distribution, sparse markets and poor road infrastructure prolong journeys and prohibit women from obtaining bean seeds (McGuire and Sperling 2016). In Kenya, this problem is being overcome by using nontraditional means of transport, such as motorbikes, to reach last-mile female farmers (Onyango 2020).

In bean breeding, the number of female scientists working on improving legume research has been reported to be limited (Ojiewo 2018b). The CGIAR datasets also showed that the number of breeders in terms of Full-Time Equivalent (FTE) was close to zero in countries such as Burundi, a manifestation of the decreased likelihood of finding even a single female bean breeder in such countries (CGIAR DIIVA 2021). This implies that even within research, there exists a gender gap in breeders who could point out that missing traits are preferred by women but that there are no differences. It should be noted that trait preferences are also strongly determined by an individual's socioeconomic and cultural situation and not just by gender (Table 3).

**Table 3:** A redefined seed system performance matrix using the reach, benefit, empower, and transform (RBET) framework. *Source*: Authors with adaptations from Maredia et al. (1999), Spielman and Kennedy (2016a) and Puskur et al. (2021)

RBET domain	Seed industry domain(s)	Indicator (units of measure)	Gender responsiveness parameters/indicators
Reach	a. Industry performance	Seed sales (metric tons) Seed packaging (unit)	Volume of seed sales by men/women/youths Seed packaging sizing and preferences by men and women
		Seed pricing (Index)	Gender seed price index Number of women/men/youths selling in different markets Gendered price discrimination on seed
		Digital platforms or apps (number)	Availability of inclusive apps and digital platform for verifying seed information
	b. Innovation	Varieties released (number)	Number of national gendered strategies/policies Perceived seed quality disaggregated by gender
		Seed security measures	Number of varieties released taking gender preferences into consideration Volume of seed accessed by women/men/youth Volume of seed accessible through noncash payments by men/women/youths Number of women/men/youths groups producing seed Number of women participation in PVS/PPB or any seed training
		Seed markets	Number of women in different seed markets Number of women and youth seed entrepreneurs Number of men/women/youths accessing seed subsidies
		Seed distribution system (formal, informal, and intermediate channels) Public vs private seed producers	Number of seed distribution channels accessible to men/women/youths Number of community seed banks serving women and youths Number of seed companies disaggregated by gender (sex, age, ownership, management etc.)
		HHI, CR4, and CR8 measures	HHI, CR4, and CR8 indices disaggregated by gender
		Seed subsidies/taxes/policies	Number of policies recognizing women in seed system/seed tax relief for women
	c. Structure	Seed value chain actors	Number of farmers growing improved varieties disaggregated by gender Number of women/men/youths accessing diverse markets Seed source disaggregated by gender Number of women/youth extension officers Number of extension services providers focusing on women and youth Number of women/youth/men trained by diverse seed actors (NARS, private, public and development partners) Number of women and youths producing seed Number of gender-inclusive seed production models Number of gendered seed dissemination models
		Seed system leadership structure	Number of men/women/youths in seed system leadership positions Number of women with decision-making capacities in community seed banks
		Capacity building	Number of women/men/youths trained on new, improved varieties Number of women/men/youths trained on seed production Number of women/youth/men trained on seed business Number of times men/women/youths recycle seed after three seasons
Benefits	a. Industry performance	Yield and variety adoption (number)	Number of women/men/youths adopting new seed varieties
		Improved income (local currency)	Number of women/men/youths with increased income Number of hours women/men/youths spend on seed access and sales Number of women/men/youths farmers using selling preferred varieties in local markets Score on seed/variety and advisory information accessible in local languages and digitally Gender seed price index
		Time use for women (hours)	Number of men/women and youths with access and control over technologies like seed planters reducing drudgery % increase in yield in women’s fields % increase in grain volume traded by women
	b. Seed registration and quality control	Existence of niche variety release and exemption system Seeds certification period	Number of gender responsive product profiles and varieties developed Number of varieties with less than 10 years of release Number of varieties which meet women’s trait preferences
	c. Innovations	R&D expenditures Community seed banks	Number of investors in women/men/youth-owned seed systems Number of women accessing community seed banks/perceived benefits Number of women/men/youths leading community seed banks
	d. Seed system governance	PVS, PPB and Tricot Demand-led Breeding	Number of women/youths benefitting from PVS, PPB and Tricot Number of women/men/youths who have gained agency and skills through PPB and PVS
		Existence of local seed producers' leadership structure	Increased participation of women and youth in seed system leadership Number of women/men/youths with increased decision-making capacities in the seed system
	e. Seed system actors	Existence of seed value chain actors including farmers, industry regulators, NGOs, private sector, gene banks	Seed supply value chain resilience Existence of green supply value chain Number of seed companies working with local and international seed distributors
Empowerment	a. Industry performance	Seed business ownership Access to seed loans	Number of seed businesses owned/led by women/men/youths Number of women/men/youths accessing seed investors
		Capacity change	Number of women/men/youths who have changed practice, attitude because of in-field demos and workshops on seed
		Community seed banks Leadership positions	Number of women/men/youths making decisions in community seed banks Number of women participating in local seed committees
		Time use	Number of women reporting reduced labor intensity/drudgery due to change in variety traits
		Number of patents	Number of varieties released with gender preferred traits Number of women breeders developing new varieties Number of patents for new innovations disaggregated by gender
	b. Intellectual property rights	Number of patents	Number of seed innovations patented by women Number of women seed breeders
	c. Digital innovations	Digital tracking of distribution systems for the last mile farmers The existence of women's seed banks Digital platforms that democratize equal access to seeds Digital access to seed credit	Digital tracking of distribution systems for the last mile farmers The existence of women's seed banks Digital platforms that democratize equal access to seeds Digital access to seed credit
	d. Mechanization	Mechanized seeding systems	Number of women/men/youths who use gender responsive seed mechanization
Transformation	a. Industry performance	Commercial seed production Seed companies' responsiveness Resilience	Number of women/men/youth-led commercial seed enterprises Number of gendered preferred seed varieties sold by seed companies Number of women/men/youths employed in seed companies Number of varieties accessed by women farmers (varietal diversity index) Replacement rate for gender preferred varieties Number of varieties accessed by women/men/youth farmers (varietal diversity index
	b. Intellectual property rights and regulations	Seed laws (number) and seed policies	Number of laws enacted to safeguard varietal preferences for women Number of policies formulated to ensure equal seed access for women Number of policies that promote regional seed trade by women/men/youths Number of seed regulations/laws and policies that are gender responsive
	c. Digital innovations	Digital tracking of distribution systems for the last mile farmers The existence of women's seed banks Digital platforms that democratize equal access to seeds Digital access to seed credit	Number of Digital Point-of-sale tracking systems that collect information which is disaggregated by gender Number of accessible seed banks to women and youths at the village-level Number of Apps tracking seed access for women Number of digital platforms offering seed credit to women/men/youths
	d. Mechanization	Mechanized seeding systems	Number of women/men/youths who use gender responsive seed mechanization
	e. Seed system resilience	Gender responsive seed system	Gendered seed system resilience index
		Climate-adaptive breeding programs Seed security	Number of gender-based food, and nutrition sovereignty for women and youth
		Agroecological resilience	Gender-aware environmentally friendly seed production system
	f. Seed system governance	Integrated seed system policies at country and international levels	Gender-aware sanitary and phytosanitary standards Biosafety standards for women and youth seed producers International treaties that regard women’s seed access rights
		Agricultural research	Existence of women farmers’ rights charter Existence of quality assurance system for women seed producers

HHI denotes Herfindahl–Hirschman Index; CR4/8 denotes four- and eight-firm concentration ratios, respectively

## Discussion of the systematic review

### Reach

In the reach domain, 10 studies from both peer-reviewed articles and gray literature, with a particular focus on common bean crops, were included. These studies are used to provide evidence on reach and participation (such as training and meetings), access to seeds and extension services, among other indicators, within the seed system (Johnson et al. 2018; Nchanji 2022). To track these participation indicators, the study mapped them to measurable indicators as proposed by Spielman and Kennedy (2016a, b). First, with regard to seed access indicators, a recent cross-country (Kenya, Uganda and Tanzania) study by Marimo et al. (2021) revealed the informal seed system to be the dominant system and was accessed by 69% of women (own seeds and seeds from neighbors). The study looks at the seed industry’s innovation and particularly focuses on indicators such as seed access. In Rwanda, it was found that women were more likely to access seeds through informal channels; thus, a more cost-effective and inclusive strategy to reach them was leveraging their social networks (Vaiknoras et al. 2019). The study highlights the seed system’s industry performance indicators, innovation, and structure. In Kenya, NGOs seem to work more with women (78%) than men (58%) in the seed system to balance the formal/improved seed access dominated by men (ibid). This implies that seeds promoted by NGOs seem to be accessed more by women because they are intentional. The seed system indicators tracked here include seed access, innovation to reach millions, and social capital, among others. In Madagascar, women participate in seed production and attend demonstration plots in the same way men do, resulting in nondiscriminatory pricing that does not favor any gender; the willingness to pay is 171% (Bosch et al. 2017). However, the study showed that, irrespective of price discrimination or the free provision of seed and farming information, the willingness to pay more for the improved seeds was low.

In Kenya, PABRA used the Point-of-Sale (PoS) app to track real-time seed distribution and found that males (62%) accessed more varieties than did their female counterparts (38%). A study also revealed that high-iron bean (HIB) varieties retail between 2.20 and 2.50 US dollars per kilogram (Onyango 2020). With regard to pricing and packaging, no gender-disaggregated data were collected; thus, the evidence of the effect of gender-based seed cost/pricing was inconclusive. Gender-disaggregated price indicators could be important for understanding price discrimination among men and women. Looking at the seed packaging indicator, it was found that packet sizes between 2 and 5 kg dominated the market. This was confirmed by an experimental Tropical Legumes II program in which 943,170 small seed packs of common bean and other legumes were sold in 13 African countries (McGuire and Sperling 2016). This implies that smaller seed packages may be preferable to smallholder farmers, especially women (Gichangi et al. 2012). On the other hand, Rubyogo et al. (2016) reported that 76.4% of farmers were satisfied with Malawi's 1.5 kg package, but no gender data are available to determine whether this approach works for men and women. Gender disaggregation was not presented in these studies, so conclusions were impossible from a gender lens.

A cross-country comparison study also revealed that women are disadvantaged in terms of access to seed information. In Tanzania, 81% of men accessed seed information from experts, whereas 53% and 56% accessed seed information from Kenya and Uganda, respectively.

Another indicator within the reach domain is the number of niche varieties/bean products tailored toward a certain group using nontraditional methods to improve seed access and delivery. The legume seed industry in Kenya and Uganda is developing rapidly, and new biofortified varieties high in iron and zinc (Nyota, Angaza and Metameta in Kenya; NAROBEAN 1, NAROBEAN 2 and NAROBEAN 3 in Uganda) have recently been released (Ugen et al. 2021). Seed access for these varieties has been enhanced for women through the Seed Credit Model (SCM) and Community Production and Marketing System (COPMAS) (ibid). While these models are yet to be scaled up, a study in Kenya found that women have dense seed networks that could be utilized to scale the models. In addition, these networks heavily rely on "informal sources" rather than men's networks (Otieno et al. 2021). This implies that even if we decide to focus only on formalized seed models, farmer-to-farmer seed exchange (informal) should be addressed within the seed system, as it is the main source/risk for misinformation. Minimizing such risks of misinformation could occur through community-led seed banks harmonized within national policies. An example is the community seed bank of Kiziba in Uganda (Wilkus 2016). This allows for the reliable transfer of seeds and other materials from the local to the formal seed sector, thus eliminating informal linkages and resulting in lower seed quality. In Ethiopia, farmer-led seed delivery organized around Local Seed Businesses (LSBs) to produce and market quality seeds that were not attractive to private companies seemed to solve the challenge of accessing seeds for women (Ojiewo et al. 2018a, b).

As proposed by Spielman and Kennedy (2016a, b), studies assessing the use of the Hirschman-Herfindahl Index (HHI), which measures firms' market shares in the seed market disaggregated by main seed/grain source for farmers, are presented in Table 4. In Kenya, the largest 4 and 8 retailers shown by the four-firm concentration (CR4) ratio (%) and eight-firm concentration (CR8) ratio (%), respectively, were 19.29% and 33.92%. Thus, the informal seed industry may be described as 'low moderately concentrated', as indicated by the HHI of 5321 in the country. The largest 4 and 8 largest wholesalers in Kenya controlled 34.76% and 59.84%, respectively, and had an HHI of 9460 (Kibiego et al. 2003). This indicates a nearly pure competitive market structure for the seeds. On the other hand, Ethiopian market concentration seems oligopolistic, as there are few value chain actors in each of the nodes, especially exporters. The HHI of the aggregators, who are at the local level, is 5582, indicating that the common bean seed/grain market is moderately concentrated, similar to retailers in Kenya (Palencia 2017). According to these analyses, women were positioned as traders in bean grains, but the seed industry was dominated by males (Kibiego et al. 2003).

**Table 4:** Concentration of common bean seeds in the market by value chain in Kenya and Ethiopia. *Source*: Author's computations based on Kibiego et al. (2003) and Palencia (2017)

Country	Value chain	Hirschman-Herfindahl Index (HHI)	Four-firm concentration (CR4) ratio (%)	Eight-firm concentration (CR8) ratio (%)
Kenya	Retailers	5321	19.29	33.92
	Wholesale	9460	37.76	59.84
Ethiopia	Aggregators	5582	55.82	–
	Wholesalers	6642	66.42	–
	Exporters	4411	44.11	–

### Benefits

In the benefits domain, the main aim is to highlight how the common bean seed system enhances the welfare of a bean farmer, especially in terms of hunger reduction, increased income, and improved resilience among women (Johnson et al. 2018; Nchanji et al 2022a, b). It is possible to quantify how these indicators improve women's welfare by measuring factors such as nutritional benefits, adoption practices, tracking time use, seed certification procedures, and community seed systems. First, adopting improved varieties is important for improving income and nutritional status. Farmers who buy seeds of improved varieties have been demonstrated to have better welfare (Walsh and Sperling 2019). An integrated impact-driven seed systems trial resulted in a 20% to approximately 68% increase in common bean adoption and improved seed production and delivery in Ethiopia (Tumsa et al. 2013). Farmers were able to obtain improved yields that resulted in surplus sales to cater to household expenditures.

In Rwanda, it was demonstrated that adopting iron-biofortified beans was successful because of women's variable preferences, with longer-lasting benefits for women (Vaiknoras et al. 2019). However, some studies indicate that males adopt improved seeds more than females do, as they have access to resources and lead in most household decision-making. This was demonstrated by Wambua et al. (2018), who also found that households in Kenya where the main decision maker was a woman had lower yields.

On the other hand, a participatory breeding program in Uganda found women groups motivated by commercial goals, resulting in the diversification of their seeds (Wilkus et al. 2018). This implies that seed systems that improve incomes are desirable and may help improve community-level nutrition. Not integrating women's roles as decision makers in seed systems may result in disproportionate resource allocation and use. A study in Kenya indicated that female household heads made fewer decisions on incomes from common bean farming using improved variety seeds (Wambua et al. 2018). As such, PPB and PVS can be avenues for motivating women who want to commercialize bean production and move out of poverty, as they include gender training modules on farm and household decision-making.

While financial welfare is important, improved nutritional status and improved cognitive abilities through bean consumption using high-iron beans have been documented (Murray-Kolb et al. 2017). Through PABRA high-iron bean breeding, access to HIB seeds may change the nutritional status of women. In Ethiopia and Uganda, women were more involved in labor-intensive activities such as weeding and threshing but had little decision to sell grains (Abera et al. 2020; Katungi et al. 2019). Studies have demonstrated that even attending training and farm demos is limited by individuals’ radius of movement and society's desire for a 'good wife/woman' (Njuguna et al. 2016).

To benefit women, the seed system should be designed to be responsive across the value chain, offer more training and extension services, and give women more autonomy in decision-making. An example is CGIAR's Excellence in Breeding program, which recognizes real-life seed choices as tradeoffs or competitive events with other livelihood farmer needs but recognizes chain-wide trait prioritization that may help overcome such tradeoffs and incentivize farmers to buy seeds (McEwan et al. 2021). It recognizes women as leading informal seed traders and local intermediaries in the seed value chain, as they sort grains as "potential seeds" (Sperling et al. 2020a, b).

### Empowerment

In this domain, we track seed system studies in common bean value chains that measure empowering actions such as women's leadership, agency, skills and factors that inherently disempower them, such as drudgery, time use, decision-making power or control over resources. Most formal seed systems involve pro-men, who are designed to attract them as farmers who own land and as household heads (Paris and Rola-Rubzen 2019). Women are not recognized formally as seed users (Brearley and Kramer 2020). Thus, gender inequalities constrain women's access to and participation in seed-related decision-making processes. In the common bean seed system in Ethiopia, 30% of the seed producers were widowed, divorced, or separated women who lacked opportunities to network or participate in community meetings that discussed seed issues (Geleta et al. 2017). This limited their participation in leadership or vying for seed-bank leadership positions. In Zimbabwe, the design of agricultural extension services, especially training workshops, was reported to be a disempowering factor, as women were excluded from distant agricultural training despite women playing a dominant role in seed processing, preservation, and storage (Matsa and Manuku 2013).

Seed systems have seen community seed banks engaging women and furthering their decision-making in accessing quality seeds. In Zambia, women were more active in variety selection at the local level (USAID/AfricaLead 2016). They also belonged to groups that made regular weekly payments that enabled them to purchase seeds. They also held leadership positions, giving them a voice in seed system activities. In contrast, in Tanzania, ownership and upper-level management of the surveyed seed companies revealed that women accounted for only 3% of the population (AGRA 2016). Women held clerical and smaller positions that did not put them in a position to voice their concerns, inherently disempowering them in seed system activities.

Apart from leadership, seed networks managed by women were found to create space for improved decision-making among women in Kenya (Otieno et al. 2021). Along the value chain, especially in terms of distribution, sparse markets and poor road infrastructure prolong journeys and prohibit women from obtaining bean seeds (McGuire and Sperling 2016). In Kenya, this problem is being overcome by using nontraditional means of transport, such as motorbikes, to reach last-mile female farmers (Onyango 2020).

In bean breeding, the number of female scientists working on improving legume research has been reported to be limited (Ojiewo 2018b). The CGIAR datasets also showed that the number of breeders in terms of Full-Time Equivalent (FTE) was close to zero in countries such as Burundi, a manifestation of the decreased likelihood of finding even a single female bean breeder in such countries (CGIAR DIIVA 2021). This implies that even within research, there exists a gender gap with fewer women who could point out the missing traits preferred by women, even though many nuances exist here, as women breeders or researchers might not tell traits pf individual women due to different socioeconomic and cultural situations that condition the choice of traits.

### Transform

Transform goes beyond just empowering women within the seed system. The aim is to create an enabling environment to identify and address gender barriers that are deeply embedded in societies, especially gender norms, inequalities, and governance structures (Cole et al. 2018; Johnson et al. 2018). Thus, studies that analyze the paradigm shift of gender barriers tuned toward optimized opportunities to realize gender equality, equity and women's empowerment are considered. According to Puskur et al. (2021), gender-transformative evidence is under researched and under profiled across all crops. However, some studies, such as Cole et al. (2018), have documented how participatory testing of postharvest technologies in Zambia led to significant gender-equal attitudes among men. It gave women control over income, made them exercise choice and voiced their concerns more freely. In southern Ethiopia, the management of small-seed enterprises was reserved for women who make all decisions regarding seeds, including sales and the management of profits (Habte et al. 2010).

Intentional digital inclusion has been proposed to overcome seed access barriers and transform the seed system (Shrader 2021). An example of the use of digital technology is the use of the point-of-sale approach by the Pan-Africa Bean Research Alliance and its partners to track seeds from seed companies to distributors (agrovets and small shops) and to use nontraditional transport methods such as motorbikes, which are cheaper and adaptable to different terrains and able to reach women farmers at the last miles (Onyango 2020). This transformative approach to the seed system would ensure that people at the last mile, especially women who incur substantial transaction costs accessing seeds, are reached.

## Discussion

### Toward gender-transformative seed system metrics and seed system policies

When seed system indicators are not put through the RBET framework and gender lens, they fall short of measuring critical seed industry characteristics. For example, while Spielman and Kennedy (2016b) provided a framework with seed system indicators with higher resolution at—spatial, social, household, farm, plot and varietal levels, the nuances in gender never came out clearly. It presented a first stab at what would need methodical re-analysis to determine industry players needs against the youth, women and vulnerable people’s needs. On the other hand, while studies such as Louwaars and De Boef (2012; and Westengen et al. (2023) have developed a holistic approach that integrates seed actors (partnerships and networks) and seed security elements, gender issues have just been employed to some extent. It is agreeable that a gender-transformative system needs institutional and technical innovations using an integrated seed system approach that involves public‒private sector partnerships and critically overcoming farmer-based seed production barriers through digital means and supply initiatives (Ojiewo et al. 2018a, b).

The current analysis takes into consideration the metrics that can inform the seed system players to engage meaningfully and address the salient gender issues such as women’s varietal preferences for certain varieties. Such analysis can be used to shape national agricultural growth strategies, set public research priorities, design private innovation incentives, construct public input provision programs, and encourage maize seed industry development and productivity.

From the evidence above, it is safe to argue that areas of action in an integrated seed system would be significantly captured in the reach domain by advocating for increased total seed production and availability. Women have increased access to better-quality seeds of common bean, even though issues such as lack of small and affordable seed packages, access to seed credit and lack of extension services seem to reduce the benefits for women and young people. Women’s empowerment through leadership in village seed banks, value chain support, PPB and PVS, and capacity development for postharvest handling, including seed and creating market linkages, are believed to springboard women from just reaching and benefiting more empowered decision making in the seed system. In Ghana, only 1 out of the 31 seed companies are led by women, while out of 92 management positions, only 3–23% are held by women, revealing low levels of gender integration within the whole seed industry (Mabaya et al. 2021). This can be overcome by national policies such as the National Seed Policy of the Republic of Ghana, the Plants and Fertilizer Act, 2010 (ACT 803), and Act 803, which seem not to address the gender aspects of women in the seed system directly. A low score for Genetic Resources reflects a lack of disclosure and corporate positions related to conserving genetic resources and benefit-sharing.

Most seed companies expressed a high level of satisfaction with the quality of seed policy instruments, including seed policy, seed acts, seed regulations, and seed strategies. However, in most cases, they also expressed a high level of dissatisfaction with the level of enforcement and implementation of these instruments. This was also the case in countries such as Ethiopia, Ghana, Malawi and Madagascar. In the worst cases (e.g., in Senegal), weak enforcement at all stages of the seed value chain leads to poor-quality seeds on the market (Mabaya et al. 2021).

The cost of seeds remains a pain-point for farmers in seed systems. A recent survey of seed companies in 13 countries revealed that the government and agro-dealers control 68% of seed sales(Agri-Experience 2018). Decentralizing such seed sales to the local level would reduce transaction costs because last-mile farmers would easily have timely and less expensive access. The fact that the cost of releasing a variety is high and averaged at US$ 3,000 in Kenya, US$ 4,000 in Mali in 2018 and US$ 27,000 in Nigeria in 2018, as posited by Mabaya et al. (2021), is a cause for concern for industry practitioners.

There have been efforts to register farmers’ varieties, which will greatly change the variety of commercialization systems (De Jonge et al. 2021). Research organizations such as the Pan-Africa Bean Research Alliance (PABRA), in collaboration with the Kenya Agriculture and Livestock Research Organization (KALRO), and private seed companies in Kenya have already taken advantage of the fast-tracked improved bean registration and release system to obtain high iron beans for smallholder farmers (Mabeya et al. 2020). These were based on the niche attributes, uniqueness and promise of solving chronic and hidden hunger within the rural farming families.

### Seed sector transformation policy

While evidence is very thin for truly gender-transformative seed systems, leveraging public policy and international treaties are possible avenues. Such policies would reduce market concentration from the hands of few players and systematically restructure the seed system for equitable and gender-aware distribution system as suggested in (Otieno et al. 2021; Sperling et al. 2022). Using metrics such as women’s networks and village-level farmer-to-farmer seed distribution systems solve the reach for the masses but also catalyze transformation from relative isolation of women and youths to being part of the seed system. What of digitally enhanced delivery systems? Reaching farmers at the last mile by bundling seeds with other agro-inputs using non-traditional methods and piggybacking on existing product supply channels or using digital point-of-sale mobile apps would be transformative and eliminate cultural barriers to seed access. A salient gender issue overcome here are the culturally limiting information access gaps that would hinder performance of the seed system. Seed companies may need to invest in these efforts and strengthen community seed banks, for example.

## Conclusions

This study sought to identify gaps in seed system metrics and highlight gender issues that need to be integrated as indicators to monitor the seed system’s performance. By examining this through the RBET framework, it emerged that gender biases exist in the seed system and very few literatures has reported on specific metrics that industry players can use. We proposed a series of metrics that integrate youth and women’s issues in the seed system.

For example, the analysis explored disparities in access to quality seed for women versus men, considering factors such as use of digital tools, seed voucher programs in the hope that these would be empowering to the women and youth within households. The study suggests public–private facing interventions that could make the seed system more efficient. For example, while policy reforms in the early 1980s have impacted seed industry growth, limitations still exist in laws that properly govern seed system relations. This means that even with DLB kind of approaches, the impact may only be partially attributable to good seed laws. With the proposed gender metrics and subsequent adoption of such or deliberate inclusion by private and public seed industry, it would be easy to enhance formulating and communicating policies that enhance seed system efficiency.

Finally, our recommendation is that thin literature exists to prove gender as part of a dynamic seed system. Therefore, use of enhanced datasets and more deliberate gender-based research is needed. On the other hand, specific interventions are needed to evaluate the community-led seed banks, provide training and extension services that are specifically designed for women farmers, and advocate for gender-responsive policies and programs that support women's participation in the seed system. It is time to take advantage of new developments, especially frameworks that cover actors within the seed system, seed system functions and seed security issues as suggested in Louwaars and De Boef (2012) and Westengen et al. (2023). However, such frameworks may also need to consider the gender nuances and framed within the Sub-Sahara African farmer needs.

In conclusion, the seed system has the potential to be a powerful tool for improving the welfare of women, youth and other vulnerable farmers, and measuring women’s indicators using the RBET framework provides gaps and opportunities to close the gender gap in the seed system by making it a more equitable and inclusive system that benefits all farmers, regardless of gender.

## Data Availability

The data are available from the authors upon request.

## Appendix

See Table 5.

**Table 5:** Author list with gender indicators they address

RBET theme	Study	Seed system indicator	Gender issue
Reach	Bosch et al. (2017)	Seed access indicators	Adoption of improved varieties and efficient distribution system for improved consumption
Reach	Kibiego et al. (2003)	Seed access indicators	Bean marketing structure was purely competitive and women-dominated with few wholesale/retail traders
Reach	Marimo et al. (2021)	Seed access indicators	Women empowerment using formal and informal seed systems
Reach	McGuire and Sperling (2016)	Seed access indicators	Inefficient distribution systems and poor transport infrastructure hinder women access
Reach	Onyango (2020)	Seed access indicators	Digital access for women
Reach	Otieno et al. (2021)	Farmer-to-farmer distribution system	Men more likely to access improved seeds than women
Reach	Palencia (2017)	Seed market concentration	Seed market dominated by men
Reach	Ugen et al. (2021)	Seed access indicators	Seed access and use of aggregators for women
Reach	Wilkus (2016)	Seed access indicators	Number of seed exchange networks
Reach	Vaiknoras et al. (2019)	Seed for nutirion	Seeds as foundation for nutrition for women
Benefit	Johnson et al. (2018)	Benefits of improved seeds	Reach, benefit and empower women for impactful gender interventions
Benefit	Walsh and Sperling (2019)	Improved cultivars	Seed pricing not favorable to women and youth
Benefit	Tumsa et al. (2013)	Benefits of improved seeds	Access to quality seeds is beneficial
Benefit	Wambua et al. (2018)	Benefits of improved seeds	Effect of access and control of productive resources including seed
Benefit	Wilkus et al. (2018)	Benefits of improved seeds	Seed exchange networks
Benefit	Murray-Kolb et al. (2017)	Benefits of improved seeds	Beans for nutrition for girls and women
Benefit	Luna et al. (2020)	Benefits of improved seeds	Beans for nutrition for girls and women
Benefit	Haas et al. (2016)	Benefits of improved seeds	Women more likely to benefit from improved seeds than men
Benefit	Abera et al. (2020)	Benefits of improved seeds	Community-level PVS and DLB for improved seed access
Benefit	Katungi et al. (2019)	Benefits of improved seeds	Using smart technologies to solve market access
Benefit	Njuguna et al. (2016)	Benefits of improved seeds	Using dialogue to benefit both men and women in a household
Benefit	McEwan et al. (2021)	Benefits of improved seeds	Evidence-based dialogue between researchers and breeders for DLB
Benefit	Sperling et al. (2020a, b)	Benefits of improved seeds	Seed security for all
Benefit	Ojiewo et al. (2018a, b)	Benefits of improved seeds	Integrated smart legume seed system empower individuals to be self-reliant—men, women and youth
Empowerment	Paris and Rola-Rubzen 2019	Gendered empowerment indicators	Fast-cooking bean attributes in seed system to empower women
Empowerment	Brealey and Kramer (2020)	Gendered empowerment indicators	Advancement in biotechnology and access to and, control and use of seeds by women
Empowerment	Geleta et al. (2017)	Gendered empowerment indicators	Seed system that caters for heterogeneity of women and design multidimensional programs that can help married women to gain full access to resources and participate in important household decision-making processes
Empowerment	USAID/AfricaLead (2016)	Gendered empowerment indicators	Women control and decision making for empowerment
Empowerment	AGRA (2016)	Gendered empowerment indicators	Integrated seed system for improved gender roles in agricultural production
Empowerment	Otieno et al. (2021)	Gendered empowerment indicators	Institutional capacity change and gendered network systems
Empowerment	McGuire and Sperling, (2016)	Gendered empowerment indicators	Seed system metrics
Empowerment	Onyango (2020)	Gendered empowerment indicators	Use of digital and modern technologies for last-mile seed delivery
